# Assessment of entropy accumulation in human subjects when exposed to low energy availability

**DOI:** 10.1016/j.heliyon.2024.e36792

**Published:** 2024-08-28

**Authors:** Cennet Yildiz, Karsten Köhler, Paulina Wasserfurth, Mustafa Özilgen

**Affiliations:** a Department of Food Engineering, Yeditepe University, Istanbul, Turkey; b Department of Sport and Health Sciences, Technical University of Munich, Munich, Germany

**Keywords:** Energy availability, Energy deficiency, Entropy generation, Lifespan estimation

## Abstract

**Background & aims:**

Adequate energy availability is essential for the body to maintain its physiological functions and achieve optimal health, especially among athletes. Unfortunately, low energy availability (LEA) is common among athletes, and it has been associated with impairments in health and performance. In contrast, an energy-restricted diet has been linked to longevity, but it is unclear how LEA affects athletes’ lifespans. The goal of the present study was to assess the impact of LEA thermodynamically on the lifespan of athletes.

**Methods:**

Data from seven healthy young endurance-trained athletes (24 ± 4 years) who underwent short-term low energy availability (125 (kJ/day) per kg FFM) once with low protein content (LEA-LP; 0.8 g/kg) and with high protein content (LEA-HP; 125 (kJ/day) per kg FFM, 1.7 g/kg), as well as a control diet (CON; 230 (kJ/day) per kg FFM, 1.7 g/kg), were used in the calculations. The athletes followed each diet for five days and expended 67.5 (kJ/day) per kg FFM. entropy generation-based thermodynamic calculations were performed based on the metabolic activity of the athletes, which was determined from oxygen consumption and carbon dioxide production rates.

**Results:**

Low energy availability was successfully induced during LEA-LP (62 ± 8 (kJ/day) per kg FFM; 95%Cl: 53–70) and LEA-HP (64 ± 8 (kJ/day) per kg FFM; 95%Cl: 56–71) diets. Despite of achieving energy deficit of −6658 ± 2110 kJ/day (95%Cl: 8609-(−) 4707) (LEA-LP), −5781 ± 623 (95%Cl: 26591-(−)4707) (LEA-HP) and excessive energy of 772 ± 1915 (95%Cl: 845-2388) (CON) statistical analyses revealed no significant differences in lifespan estimations among diets (CON: 72 ± 8 years (95%Cl: 65–79), LEA-LP: 74 ± 7 years (95%Cl: 68–80), and LEA-HP: 73 ± 11 (95%Cl: 62–83)).

**Conclusions:**

This study suggests valuable insights into the intricate relationship between energy availability, entropy generation, and lifespan among athletes. Whereas entropy generation levels and the lifespan of athletes remained stable depending on diets, the distinguished differences in energy deficiency and energy availability underline the need for a profounder investigation of underlying physiological mechanisms to improve the health and performance of athletes.

## Significance of the study

• “Entropic age theory” suggests that a living system dies after accumulating a certain amount of entropy.
• Understanding of the underlying mechanisms governing metabolic adaptation in response to energy deficiency and its potential implications for entropy accumulation and estimated lifespan is crucial to optimize the conditions for athletes.
• By employing thermodynamic assessments, this study pioneers a predictive framework for estimating athlete lifespan without compromising individuals to prolonged, potentially harmful low energy availability conditions. This predictive capability of the biothermodynamic approach holds immense promise in adapting personalized diets and exercise plans to improve athletes' health and performance over their lifespans.

## 1 Introduction

A cornerstone of sports nutrition is meeting energy needs and preventing disease and bodily injuries while improving performance. An adequate energy intake relative to the expenditure of training and competition protects the body from energy deficiency while avoiding the accumulation of excessive body fat stores [1]. Athletes should consume adequate amounts of carbohydrates, lipids, and proteins in addition to fluids, vitamins, and minerals in order to fuel their training as well as their primary energy requirements [2]. The energy demand of athletes is affected by the training period, competition cycle, and exercise intensity. Exposure to heat or cold, stress and some physical injuries also affect energy requirements.

Energy availability (EA) is critical to sustaining the body's physiological functions, especially for athletes. EA can be determined by subtracting the exercise energy expenditure (EEE) from the dietary energy intake (EI) as a function of FFM [3]. Regarding bioenergetics, the EA concept posits that after the energy demands of exercise have been met, the remaining dietary energy intake is available for other physiological functions such as thermoregulation, growth, cell maintenance, reproduction, and immunity [4]. Consequently, a state of low energy availability (LEA) can result from suboptimal dietary EI, such as in the form of caloric restriction (CR), high levels of exercise energy expenditure, or a combination of both in athletes. In the state of LEA, insufficient energy is available for maintaining physiological functions and optimal health in the body [5]. For an athlete, LEA can trigger metabolic and hormonal responses, such as reduced leptin secretion, suppressed hypothalamic-pituitary-thyroid and hypothalamic-pituitary-gonadal axes, reduce bone formation, increased bone reabsorption, and decreased skeletal muscle protein synthesis [6].

**Fig. 1:**
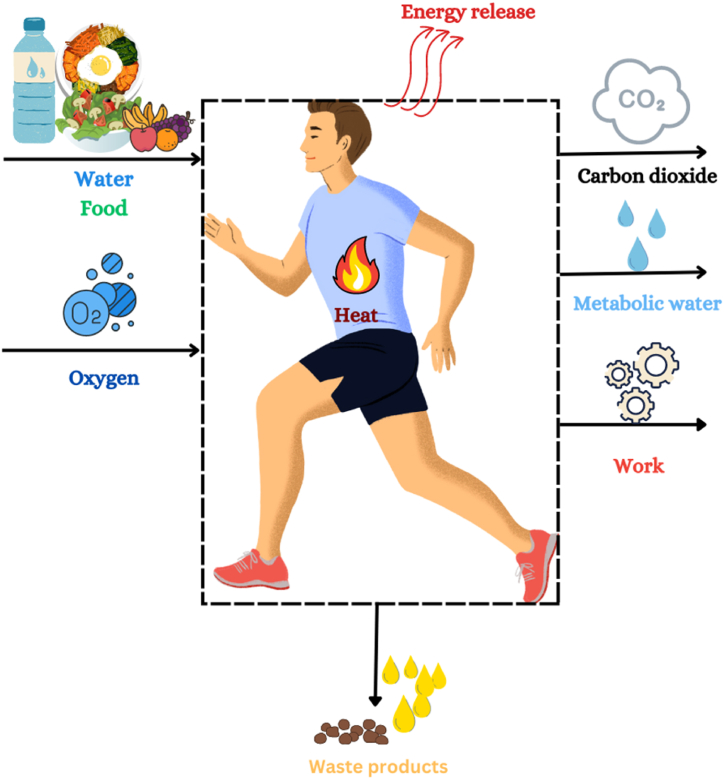
Schematic description of the athlete's body as a thermodynamic system.

Caloric restriction (CR) is one of the primary pathways toward LEA [5] it is often associated with compromised health and performance. The studies have suggested that CR extends lifespan [[7], [8], [9], [10], [11]]. However, to our knowledge, no study in the literature has explored its impact on an athlete's longevity. Exploring biothermodynamics can explain the connection between energy availability and ageing. Fig. 1 demonstrates an athlete ‘s body from a biothermodynamic perspective**.**

### 1.1 Thermodynamics of Athlete's body

Biothermodynamics, within the territory of aging and longevity studies, explores the intricate balance between energy transformations, entropy generation (S_gen_), and the lifespan of biological systems [12]. Erwin Schrödinger's influential book, “What is Life?” (1944), initiated a thought-provoking discourse on understanding life through the lens of thermodynamics. Schrödinger investigated the fundamental principles of physics and thermodynamics to contemplate. Following this path, Prigogine and Wiame (1946) [13] maintained this investigation of thermodynamics and its relationship with life. They stated that living organisms sustain their presence far from equilibrium. Based on these studies, the amount of research in the biothermodynamics field has recently increased. Lenord Hayflick suggested that modern theories converge on the centrality of molecular structure and function changes in ageing, aligning with the interpretation of the “Second Law of Thermodynamics” [14]. entropy, defined as the dispersion of concentrated energy, tends to increase when unhindered due to the relative strength of chemical bonds formed by activation energy. Silva and Annamalai (2009) [9] and Anamalai and Silva (2012) [15] investigated the effects of diet and exercise on the lifespan of the human by calculatingentropy generation (S_gen_) rate throughout the lifespan. Kuddusi (2015) [16] developed a thermodynamic model to predict the lifespan of the people who live in different regions of the Türkiye. Özilgen's and his biothermodynamic research group studied the effect of diet composition, calorie restriction and diseases on the S_gen_ rate and estimated lifespan [10,11,[17], [18], [19], [20], [21], [22], [23], [24], [25], [26], [27], [28], [29], [30], [31], [32], [33]]. These studies also contribute to the understanding of interactions of nutrient metabolism, energy transformation, and S_gen_.

**Fig. 2:**
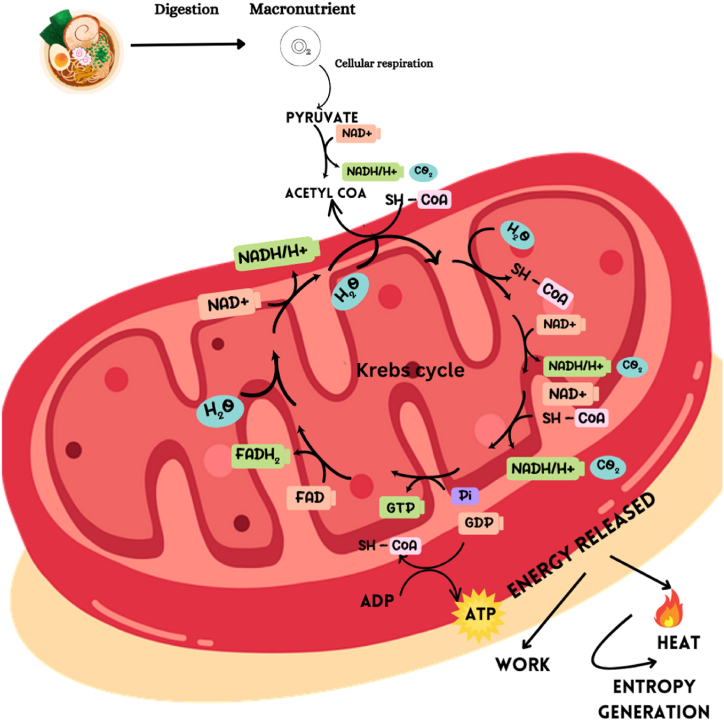
Cellular metabolism. (During the metabolism of macronutrients, energy is released, and part of energy is converted into work (stored as free energy within ATP) and reminder is converted into heat (causing entropy generation in the body)).

At the cellular level, metabolic processes drive energy conversion in living organisms, facilitating essential functions like maintaining core body temperature and powering muscular activity [34] (refer to Fig. 2). Catabolism, and anabolism are essential metabolic processes for cellular survival, growth, development, and reproduction. Energy transformation within cellular systems follows the “First Law of Thermodynamics”, where macronutrients/food serve as external sources of energy (see Fig. 1). Carbohydrates, lipids, and proteins store chemical energy in their bonds. During their catabolism, energy is harvested through a series of oxidation reactions (Fig. 2) driven by the “Second Law of Thermodynamics.”

The biological system functions are similar to a bio-engine that converts chemical energy from food into chemical energy stored in ATP molecules, acting as “work currency” akin to the way muscle work drives metabolism ([9]). In metabolic reactions, only a fraction of the energy is used to perform work. The other part of the energy is lost as heat (random energy) or entropy is generated Therefore, thermodynamic entropy can be viewed as a measure of the energy that does not exist to perform helpful work [35]. Unlike S_gen_, thermodynamic entropy is a property. Entropic age is measured in terms of S_gen_. This entropy is continuously flushed out by the body through heat loss, sweating and nasal exhaust, just like in an irreversible steam turbine where S_gen_ is flushed out through exhaust but maintaining the turbine at a steady state {dS/dt = 0 where S = ms, m, mass of steam within turbine) and entropy is a property of steam; steady state implies steady T and P and hence steady "s" and hence dS/dt = 0}. If generated entropy is not flushed out from the body, the entropy of the cell, a property increases or cell temperature increases.

The concept of “entropic age” aids in understanding ageing-related changes, such as cellular function decline and system breakdown. All living organisms exist far from equilibrium, relying on energy intake and output to manage entropy. When the body fails to export entropy, it accumulates internally, causing damage and aging [8]. If entropy accumulation surpasses the body's tolerance, the biological system cannot survive [14,25,26,35,36].

Sports and increased muscular activity escalate the body's energy demands, necessitating a higher nutrient intake to fuel these needs. As carbohydrates, amino acids, and fats undergo metabolism in the presence of oxygen during physical activity, the resulting heat dissipation amplifies (see Fig. 1, Fig. 2). This metabolic process is accompanied by the generation of entropy, a crucial factor in the functioning and aging of living organisms.

Energy metabolism plays a crucial role in various biological activities, from growth and reproduction to muscle work performance. Hormone-level control mechanisms regulate energy uptake and allocation for different bodily functions, balancing anabolism and catabolism cycles [37]. Energy deficiency and low energy availability, resulting from energy restriction and energy expenditure, alter the balance between anabolic and catabolic metabolism [38], distributing energy homeostasis. As many as 3 days of LEA (<30 kcal/kg/FFM) were shown to lead to metabolic and endocrine dysregulation, which are a consequence of the human body's natural response when exposed to an energy deficit (Loucks et al., 1994)^1^. The observed metabolic and endocrine changes include but are not limited to, a marked decline in triiodothyronine, lower fasting blood glucose and insulin levels, and impaired muscle protein synthesis [39].

This disturbance may affect metabolic responses, altering dissipated heat levels and, consequently, the entrop accumulation rate. These changes could potentially affect an athlete's longevity.

The primary aim of this study is to comprehensively assess and quantify the impact of LEA on athletes’ lifespans using thermodynamic analysis. By examining the metabolic and energy-related parameters in athletes subjected to varying levels of energy availability through controlled diets, this research seeks to elucidate the complex connections between energy intake, entropy generation, and its potential impacts on the estimated lifespans of athletes.

## 2 Materials and method

### 2.1 Study design

This study was designed to evaluate athletes' bodies in terms of thermodynamics depending on their energy availability using the data of Murphy et al. *'s* (2021) [40] randomized, single-blind, repeated measures crossover pilot study [40]. The study received approval from the University of Nebraska—Lincoln's Institutional Review Board (IRB#15895) on March 17, 2016. It was also registered at www.clinicaltrials.gov (NCT02945410; accessed on December 19, 2019). In this study, data from seven young, healthy, endurance-trained men were used to reanalyze the study of Murphy et al. (2021) [19]. The characteristics of the subjects are presented in Table 1.

**Table 1:** Physiological characteristics of the athletes.

**Age (y)**	23.8 ± 4.1
**Height (cm)**	181.5 ± 9.5
**Weight (kg)**	86.9 ± 7.6
**Fat Mass (kg)**	15.2 ± 3.1
**Fat-Free Mass (kg)**	69.5 ± 7.6

The subjects consumed three different diets during the experiments in a randomized order: The control diet (CON) provided 230 (kJ/day) per kg FFM and a protein intake of 1.7 g/kg/day. In the two intervention diets, which resulted in LEA, dietary energy intake was restricted to 125.5 (kJ/day) per kg FFM, once with a low protein intake (0.8 g/kg/d; LEA-LP) and once with a high protein intake (1.7 g/kg/d; LEA-HP). During all conditions, participants exercise to expend 63 (kJ/day) per kg FFM, resulting in energy availability of 167 (kJ/day) per kg FFM (CON) or 63 (kJ/day) per kg FFM (LEA). In this study, we reduced energy availability to 63 (kJ/day) per kg FFM based on the previous research [20,21] showing that 5 days of EA ≤ 125 (kJ/day) per kg FFM energy availability caused negative changes in endocrine and metabolic responses and the uncoupling of bone in reactive young women [41,42]. In the study, the participants were randomly assigned to one of six condition sequences through the use of a random number generator. Following this, they underwent a washout period of at least two weeks between conditions, during which they maintained their typical exercise and dietary habits. Notably, this washout period exceeded the previous 10-day period to restore body weight and metabolic hormones after brief exposure to an LEA of 63 kJ kg FFM^−1^·day^−1^. The study design is demonstrated in Fig. S1.

### 2.2 Diet preparation

In this study, clinical products- Ensure Plus High Protein from Abbott Nutrition in Chicago, IL, USA, along with maltodextrin from Tate and Lyle in London, UK were utilized. The LEA-LP diet incorporated Ensure Plus to provide 0.8 g kg BW^−1^ protein, supplemented with maltodextrin to reach a caloric intake of 125.5 (kJ/day) per kg FFM. In the other conditions, maltodextrin consumption was matched with LEA-LP, and the required quantities of both clinical products were calculated to attain 1.7 g kg BW^−1^·day^−1^ and either 125.5 kJ. kg FFM^−1^·day^−1^ (LEA-HP) or 230 kJ kg FFM^−1^·day^−1^ (CON).

Participants consumed maltodextrin dissolved in 800 mL of water·hour^−1^ during daily exercise bouts, supplemented with 1.2 g sodium chloride·L^−1^ to prevent dehydration and improve palatability. For accurate intake measurement and participant blinding, a wholly liquid diet from products used in previously employed products was provided. The dietary volume across conditions was standardized through dilution with water. Participants were instructed to consume their food in at least three meals spread throughout the day, and each participant consumed a consistent number of meals every day throughout the study. While participants were allowed to consume non-caloric beverages, they were requested to record their consumption [19].

During the study, participants were supplemented with calcium and vitamin D intake, including washout periods, to standardize differences. The quantities administered in each condition were adjusted to compensate for any deficit from the highest amount provided during the entire study [19].

### 2.3 Daily exercise prescription

The daily aerobic exercise sessions on the cycle ergometer were tailored to expend 63.3 (kJ/day) per kg FFM at the power output that corresponds to 60% of the peak oxygen uptake achieved during the preliminary graded exercise test. The duration of the daily exercise sessions was calculated by dividing the target energy expenditure (EE) rate of the exercise session by the EE at the determined power output. Any additional exercise or intense physical activity was not allowed. Compliance was measured by using a waist-worn accelerometer (ActiLife G3TX+, ActiGraph, Pensacola, FL, USA).

### 2.4 Energy balance calculations

Thermodynamic energy balance calculations were performed based on macronutrient metabolism [16]. Equations (1), (2), (3) belong to the chemical reaction of oxidation of the macronutrients. Table 2 presents individuals’ macronutrient intake, oxygen consumption, and carbon dioxide production rates for each diet. The substrate oxidation rates were determined via Equations (1)–(3) to perform thermodynamic calculations [43]. Then, a macronutrient balance was determined.

(1) $$\text{Fats}\;FOx\left(g/d\right)=1.67VO_{2}\left(L/d\right)\;‐\;1.67VCO_{2}\left(L/d\right)\;‐\;0.307Pox$$

(2) $$\text{Carbs}\;CHOx\left(g/d\right)=4.55VCO_{2}{\left(L/d\right)}_{-}\;3.21VO_{2}\left(L/d\right)+0.459Pox$$

(3) ProteinsPOx(g/d)=[Restingmetabolicrate(kJ/d)x0.15]/16.74(kJ)

**Table 2:** Daily macronutrient intake, oxygen consumption and carbon dioxide production rate.

	Protein Intake (g/d)	Protein Mass (%)	CHO Intake (g/d)	CHO Mass (%)	Fat Intake (g/d)	Fat Mass (%)	VO_2_ (L/d)	VCO_2_ (L/d)	RQ
**CON**	147 ± 13	3.81 ± 0.25	613 ± 88	15.12 ± 1.06	112 ± 11	2.84 ± 0.08	721 ± 80	631 ± 80	0.88 ± 0.1
**LEA-LP**	69 ± 6	3.32 ± 0.21	345 ± 49	15.77 ± 0.57	59 ± 5	2.78 ± 0.11	704 ± 83	557 ± 73	0.80 ± 0.07
**LEA-HP**	136 ± 31	6.31 ± 1.41	280 ± 29	12.69 ± 0.34	27 ± 5	1.39 ± 0.39	731 ± 105	611 ± 74	0.84 ± 0.005

It was assumed that 15% of the resting metabolic rate (RMR) is provided by the oxidation of protein [43]. In the equations, VO_2_ represents the oxygen consumption rate, and VCO_2_ defines the carbon dioxide production rate. Here, fat, carbohydrate (CHO), and protein oxidation were calculated for daily oxidation by taking into account how much O_2_ consumed and CO_2_ produced during exercise, non-exercise activity, and resting. Macronutrient metabolization level per day was computed by using O_2_ consumption and CO_2_ production data for exercise, resting, and non-exercise activity.

Equations (4), (5), (6) show the macronutrient oxidation reaction. Glucose, palmitic acid, and the average amino acids represent carbohydrates, lipids, and proteins, respectively. The heat and work rates for each diet were calculated based on these biochemical reactions. The calculations are based on the metabolism of glucose, palmitic acid, and average amino acids since it would be impractical to include every carbohydrate, lipid, and protein of the nutrients in the calculations for this purpose. Therefore, the 80%-20% rule, which aims to get 80% of the modelling benefits within 20% of the difficulty level, was employed in this study [15]. Accordingly, Equations (4), (5), (6) were employed to represent the metabolic reactions of carbohydrates, lipids, and proteins.

(4) *C*_6_*H*_12_*O*_6_ + 6*O*_2_ →6*H*_2_*O* + 6*CO*_2_

(5) *C*_*1*6_*H*_32_*O*_2_ + 23*O*_2_ →16*CO*_2_ +16*H*_2_*O*

(6) *C*_4.57_*H*_9.03_*N*_1.27_*O*_2.25_*S*_0.046_ +*5.7025O*_*2*_ →4*.515H*_2_*O* + *4.57CO*_2_ + *0.023S*_*2*_

Equations (4), (5), (6) above were used to simplify the metabolic reaction. During training, lactic acid accumulates in the athlete's muscles, and then it is reused. The accumulation and reuse of lactic acid do not affect the energy extracted from carbohydrates, such as enthalpy. Enthalpy remains unaffected because it is a state function, dependent only on the initial (carbohydrates and oxygen) and final (carbon dioxide and water components). Therefore, entropy generation during the lactic acid utilization and reuse was neglected [44]. In this study, the group contribution method was used. Thermodynamic properties are not found easily in the table due to complicated bio-reactions. Therefore, it is necessary to calculate the h and s values of chemicals using the group contribution method developed by Joback and Reid (1987) [45]. According to this method, a complicated biological structure is broken down into its subunits, and then its thermodynamic properties are evaluated for each subunit. The thermodynamic properties of the complex chemical structure are found by summing the thermodynamic properties of its subunits. The indigestible chemicals in the food are not included in the calculation.

In this study, we followed the procedure defined by Kuddusi and modified some parts of the procedure. It was assumed that nutrient intake and O_2_ in the body were at 25°C, and H_2_O and CO_2_ left from the body at 37 °C; the quantity of metabolic energy release rate was calculated by Eq. (7).

(7) $$\Delta Ḣ=\sum \dot{n_{p}}{\left(h_{f}^{-{}^{\circ}}+h^{-}-h^{-{}^{\circ}}\right)}_{p}-\sum \dot{n_{r}}{\left(h_{f}^{-{}^{\circ}}+h^{-}-h^{-{}^{\circ}}\right)}_{r}$$

where $\Delta Ḣ\Delta \dot{Q}$ implies the enthalpy or heat produced by the body, $\dot{n_{p}}$ and $\dot{n_{p}}$ represent the mole number rates of the products leaving the body and the reactants entering the system, respectively $h_{f}^{-{}^{\circ}}$, $h^{-}$ and $h^{-{}^{\circ}}$ define the enthalpy, enthalpy at reaction temperature and enthalpy at the standard condition. The thermodynamic properties of nutritional elements and the waste are given in Table 3. Silva and Annamalai (2008) [36] suggest the oxidation of 1 mol of each of glucose; palmitic acid and the average 20 amino acids lead to the production of 32, 106 and 8 mol of ATP, respectively. Total work achieved depending on the formation of the ATP molecules was determined by Equation (8); 34.6%, 32.2% and 10.4% present the metabolic efficiency (η) of glucose, palmitic acid, and the average of 20 amino acids, respectively [36,46].

(8) $${\dot{W}}_{ATP}=\sum ŋ\dot{\Delta H}$$

where, ${\dot{W}}_{ATP}$ implies the total work performance rate by ATP utilization. The energy consumed with food converts into heat and work after metabolization. Energy is allocated to be converted on thermal energy and perform work. Work performance can be explained the capacity of a system to do useful work. Work performance is divided into internal and external work performance. Internal work performance defines unobservable work such as the heart pumping, breathing, digestion, muscle contraction and relaxation, and sending and receiving chemical and electrical signals throughout the body. External work performance implies the part of observable muscle work such as running, cycling, and movement of body parts [17,47]. External work performance equals the sum of EEE and Non-Exercise Activity Thermogenesis (NEAT) (represented in Table 4).

**Table 3:** Thermodynamic properties of the nutrients and the products of the metabolism at 1 atm (adapted from Ref. [16]).

	Enthalpies and entropies of the macronutrients, O_2_, CO_2,_ and H_2_O at 1 atm			
**chemical**	$h_{298K}^{-}$**(kJ/kmol)**	**S**_**298K**_**(kJ/kmol K)**	$h_{310K}^{-{}^{\circ}}$**(kJ/kmol)**	**S**_**310K**_**(kJ/kmol K)**
C_6_H_12_O_6_ (glucose)	−1260 × 10^3^	212	–	
C_16_H_32_O_2_ (palmitic acid)	−835 × 10^3^	452	–	
C_4.57_H_9.03_N_1.27_O_2.25_S_0.046_ (average of the 20 amino acids)	−385 × 10^3^	1.401x119	–	
O_2_	8682	218		220
H_2_O			10,302	219
CO_2_			9807	243

**Table 4:** Daily energy intake, resting metabolic rate, and exercise energy expenditure.

	EI (kJ/d)	RMR (kJ/d)	NEAT (kJ/d)	DIT (kJ/d)	EEE (kJ/d)	Req EE
**CON**	16593 ± 1846	7702 ± 889	1765 ± 787	1659 ± 185	4769 ± 662	15649 ± 1609
**LEA-LP**	9041 ± 1008	7225 ± 658	1839 ± 938	905 ± 101	4612 ± 1866	14722 ± 868
**LEA-HP**	9050 ± 1008	7275 ± 1180	2795 ± 243	905 ± 101	4774 ± 828	15659 ± 2568

Total heat loss that occurs due to evaporation via perspiration and respiration was determined via Equation (9) with the conversion rate suggested by Hall and Hall (2020) [32,48], by following the method of Öngel et al. (2021) [32]. We assumed that water produced as a result of macronutrient metabolism was taken away from the body by perspiration and respiration. Heat transfers due to radiation, and convection were not taken into account because our calculations were based on the metabolic process of macronutrients.

(9) $${\dot{Q}}_{total}=3{Ẇ}_{ATP}$$

In Eq. (10), ${\dot{Q}}_{total}$ represents the total heat loss from the body due to evaporation, *Ẇ*_*ATP*_ indicates the total work performance rate by ATP utilization. Athletes are living organisms that are capable of performing work with the energy released when ATP is converted into ADP, as explained by Öngel et al. (2023) [10].

Table 3 represents enthalpy, enthalpy at reaction temperature and enthalpy at the standard condition and entropies for the combustion of glucose, palmitic acid and the average of 20 amino acids in the human body, as per Equations (4), (5), (6)), respectively [16]. Total energy expenditure (TEE) includes resting metabolic rate (RMR), non-exercise activity thermogenesis (NEAT), dietary-induced thermogenesis (DIT), and exercise energy expenditure (EEE) [49]. The RMR of each athlete was measured using indirect calorimetry with measurement of VO_2_ and VCO_2_. In indirect calorimetry, energy expenditure is calculated from nutrient metabolism by measuring oxygen consumption, and carbon dioxide production. Indirect calorimetry was also used to measure EEE, and a wrist-worn triaxial accelerometer determined NEAT (data represented in Table 4). Energy balance and energy availability (see Figure SI 1 and Fig. S2) for each diet were calculated by using the data in Table 4. Here, it was assumed that DIT equals 10% of EI. Energy availability (EI –EEE) and energy balance (EI-TEE) were deficient and calculated using data represented in Table 4.

### 2.5 Entropy balance

**Fig. 3:**
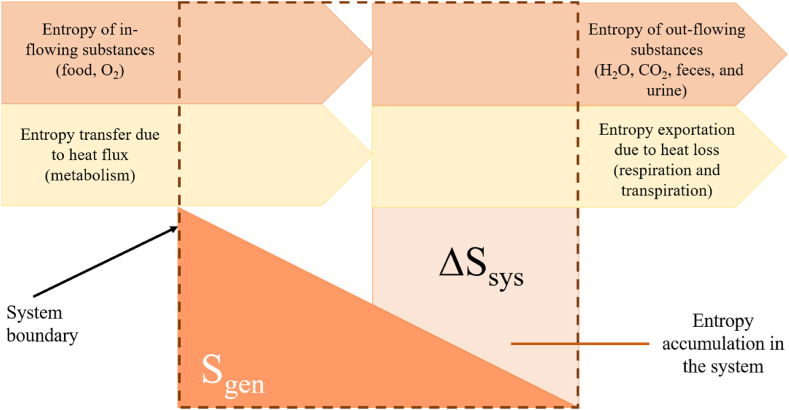
The entropy balance within a human body system, highlighting the interplay between entropy generation, entropy exportation, and entropy accumulation.

In this study, it was assumed that athletes consumed these diets throughout their lifespans and that the entropy generation rate was higher than entropy exportation because the human body does not work with 100 % efficiency and accumulates entropy. Entropy generation and accumulation calculations are performed based on these assumptions. The entropy generation rate due to the oxidation of macronutrients was calculated by using Equation (10).

(10) $$\sum_{in}{\left[\dot{m\;}s\;\right]}_{in}-\sum_{out}{\left[\dot{m\;}s\;\right]}_{out}-\sum \frac{\delta \dot{Q}}{T_{b}}+\sum {\dot{S}}_{gen}-\sum {\dot{S}}_{\exp}=\frac{{d\left[S\right]}_{system}}{d_{t}}$$

where, $\frac{{d\left[\;m\;s\right]}_{system}}{dt}$ is the rate of entropy accumulation and should be integrated to evaluate structural impairment within the system. $\sum_{in}{\left[\dot{m}\;s\right]}_{in}$ and $\sum_{out}{\left[\dot{m}\;s\;\right]}_{out}$ are the entropy rate inflow and outflow entropy through the system boundaries. $\sum_{i}\frac{\delta \dot{Q}}{T_{b}}$ is the rate of the entropy transfer with the heat released from the system. $\dot{Q}$ is the differential amount of the heat transfer rate, which is not available for doing work (it only represents the heat transfer occurring due to metabolism). Therefore, it does not include the heat transfer due to radiation and convection); $\sum {\dot{S}}_{gen}$ implies the entropy generated within the system, including ${\dot{S}}_{gen}$ due to glycogen and lipid reuse, and T_b_ is the temperature of the human body at the boundary. In this study, body temperature T_b_ was 37°C. Eq. (11) was used to calculate entropy generation due to the reuse of glycogen and fat. $\sum {\dot{S}}_{\exp}$ implies the total entropy exportation rate with faeces, methane, urine, and heat loss due to evaporation., it was calculated via Eq. (12). Fig. 3 represents schematically the entropy balance equation within the system boundaries. Equation (10) includes the calculations based on the macronutrient reactions presented by Equations. (4), (5), (6)) [16].

According to Ulu et al. (2021) [11], storage of glycogen and lipids causes negligible entropy generation, while glycogen and lipid reuse lead to entropy generation. Entropy generation due to glycogen and lipid reuse was found by Equation (11).

(11) $$S_{\text{gen}}\;\left(\text{kJ}/\text{kg}‐K\right)=\left(\text{Amount}\;\text{of}\;\text{glycogen}\;\text{reuse}\right)\;\left(1.25\;\text{kJ}/\text{mol}‐\text{Kl}\right)\;\left(1\;\text{mol}/180\;g\right)+\left(\text{Amount}\;\text{of}\;\text{lipid}\;\text{reuse}\right)\;\left(1.93\;\text{kJ}/\text{mol}‐K\right)\;\left(1\;\text{mol}/256\;g\right)$$

The entropy exportation rate was calculated by assuming that 5.8 % of energy intake was lost in faeces, 0.4 % of energy intake was exported as methane and 4.5 % in the urine (Equation (13)) [50]. Total heat loss data are represented in Table S3.

(12) $$S\_\left(\exp\right)=\left[\left(EI\;x\;0.058\right)+\left(EI\;x\;0.004\right)+\left(EI\;x\;0.045\right)+{\dot{Q}}_{total}\right]/T$$

Entropy generation was calculated with Equation (10) by using the procedure of Kuddusi (2015) [16]. Entropy generation and entropy exportation values are shown in Table S4. It is known that some fraction of entropy generation is exported from the body with waste products and CO_2_ and H_2_O. Therefore, entropy accumulation should be calculated to predict the lifespan of the body.

The lifespan entropy accumulation limit was 11,404 kJ/K kg, and the lifespan was estimated for each athlete [9,18]. In this study, an athlete's lifespan was calculated based on entropy accumulation because entropy accumulation in the biological system causes structural impairment and disorder in the body. In the research done by Silva and Annamalai [9,18], they assumed that all generated entropy by the human accumulates in the system. The calculations were performed for three different situations to predict the effects of diets on lifespan. In the first condition, it was assumed that athletes consumed all diets throughout their lifespans. For the second and third states, it was considered that athletes followed the LEA diets for 5 and 10 years, then continued the CON diet until their 50 (retirement) and, after retirement, followed the new diet (see Table S5) (prepared by Yildiz et al., 2022 [22]).

(13) $$Lifespan=\frac{\;Lifespan\;entropy\;accumulation\;limit\;\left(11,404\;kJ/K\;kg\right)\;}{Accumulated\;entropy\;within\;the\;body\;\left(\frac{kJ/\;K\;kg}{s}\;\right)\;}\;x\;\frac{1\;year}{\left(365\;x\;24\;x\;60\;x\;60\right)s}$$

### 2.6 Statistical analysis

All statistical analyses were performed by using SPSS software 28.0 (IBM, New York, USA). Descriptive statistics involves a comparison of entropy generation rate and lifespan estimation among three different diets. The distribution of data was determined through Shapiro tests and histograms. Given that all data was non-normally distributed, differences among the diets in terms of macronutrient oxidation, work performance, entropy generation, and lifespan estimation were tested by Kruskal-Wallis (1952) [51]. In the current study, the p-value <0.05 represents statistical significance. For the determination of the difference between % energy released form carbohydrates and fat, Related-Samples Wilcoxon Signed Rank Test.

## 3 Results

### 3.1 Macronutrient metabolism

**Fig. 4:**
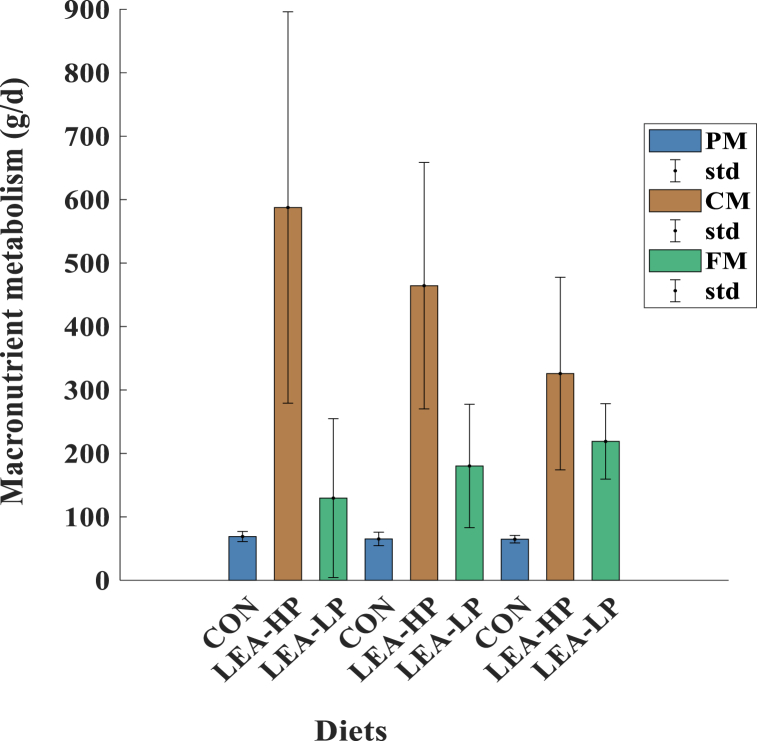
Graph of percentage of daily macronutrient metabolization during each diet (Calculation was performed based on macronutrient intake. CON: control diet, LEA-LP: low energy availability diet with low protein content, LEA-HP: low energy availability diet, PM: protein metabolization rate (g/d), CM: carbohydrate metabolization (g/d), FM: fat metabolization rate).

Our results showed that metabolized carbohydrate amount was greater during CON (587.59 ± 308.40 g/d, 95%Cl: 302.36–872.82) when compared to LEA-LP (325.81 ± 151.80 g/d, 95%Cl: 185.41–466.21; p = 0.043) and did not differ from LEA-HP diet (464. 34 ± 194.17, 95%Cl:284.77–643.92; p = 0.491). Daily fat metabolization rates were 129.60 ± 125.29 g/d (95%Cl: 13.72–245.48), 218.91 ± 59.48 g/d (95%Cl:1763.90–273.92), and 180.23 ± 97.28 g/d (95%Cl:90.26–270.20) for CON, LEA-LP, and LEA-HP, respectively and did not differ from each other (p > 0.050). Fig. 4 presents the percentage of macronutrients metabolized/oxidized by the athletes. The percentage of protein metabolization rates were similar among diets; LEA-LP (64.75 ± 5.90 (95%Cl:59.30–70.21), CON (69.04 ± 7.97 (95%Cl: 61.67–76.41), LEA-HP (65.20 ± 10.58 (95%Cl:59.30–70.21)) (p > 0.050).

Consequently, glycogen reuse was higher during LEA-HP consumption than the LEA-LP (p = 0.015). Contrary to expectations, lipid was reused during the consumption of the CON diet for three individuals (see Table S1). However, among the diets, there was no significant difference in terms of lipid reuse (p > 0.050).

As presented in Table 5, Athlete's body released much more energy from carbohydrates during exercise when compared to fat (p < 0.001). This difference was higher for CON. However, the energy released from carbohydrates and fat among the diets did not differ significantly (p > 0.05).

**Table 5:** % Energy released from carbohydrates (CHO) and fat during exercise.

	Energy release from CHO (%)	Energy release from fat (%)
CON	72.60 ± 14.96 (%95 Cl: 58.77 ± 86.43)	27.40 ± 14.96 (%95 Cl: 13.57 ± 41.23)
LEA-LP	59.81 ± 7.50 (%95 Cl: 52.88 ± 66.75)	40.18 ± 7.50 (95 % Cl: 33.25–47.11)
LEA-HP	61.66 ± 8.75 (95 % Cl: 53.56 ± 69.75)	38.34 ± 8.75 (95 % Cl: 30.25 ± 46.44)

There was no glycogen reuse during exercise for each case. Therefore, Table 6 includes only lipid reuse data for the cases. When athletes consumed the CON diet, lipid reuse was not observed during exercise. During exercise, lipid reuse was significantly higher in the athletes following the LEA-HP diet compared to those consuming the LEA-LP diet (p = 0.002).

**Table 6:** Lipid reuse during exercise.

	Lipid reuse (g)
CON	0
LEA-LP	2.07 ± 5.38 (%95 Cl: 2.90-7.05)
LEA-HP	20.55 ± 8.18 (%95 Cl: 12.98–28.11)

### 3.2 Energy balance, energy availability, and work performance

Our results showed that both LEA diets led to a sustained energy deficit (p < 0.001: LEA-HP vs. CON, p = 0.006: LEA-LP vs. CON; see Table 7), but the energy deficit did not differ between LEA-LP (−5672 ± 687 kJ/d (95%Cl: 6308-(−)5036) and LEA-HP diets (−6609 ± 2113 kJ/d kJ/d (95%Cl: 8564-(−)4655); p = 0.413).

**Table 7:** Energy balance and energy availability.

	EB (kJ/d)	EA [kJ.kg FFM^−1^day^−1^]
**CON**	943 ± 1806 [95%Cl: 726-2614]	169 ± 10 [95%Cl: 160–198]
**LEA-HP**	−6609 ± 2113 [95%Cl: 8564-(−)4655]	64 ± 8 [95%Cl: 56–71]
**LEA-LP**	−5672 ± 687 [95%Cl: 6308-(−)5036]	62 ± 9 [95%Cl: 53–70]

Energy availability was reduced during LEA-LP (61.5 ± 9.2 kJ kg FFM^−1^ (95%Cl: 53–70)) and LEA-HP diet (64 ± 8 kJ kg FFM^−1^ (95%Cl: 56–71)) when compared to CON (169 ± 10 kJ kg FFM^−1^ (95%Cl: 160–198); p < 0.003; see Table 7).

Work performance was determined by calculating metabolic heat production and efficiency of metabolism values (see Table S2). As shown in Table 8, following the low energy availability diet caused lower work performance when compared to the CON diet. However, there was no statistically significant difference between the work performance for all diets (CON-LEA-HP: p = 0.121, CON versus LEA-LP: p = 0.093, LEA-HP versus LEA-LP: p = 0.897).

**Table 8:** The work performance for each diet.

	Work performance (kJ/d)
**CON**	4911 ± 954(95%Cl: 4029–5793)
**LEA-LP**	4108 ± 450(95%Cl: 362–4525)
**LEA-HP**	4170 ± 550(95%Cl: 3611–4692)

### 3.3 Entropy accumulation and lifespan estimation of the athletes

According to statistical analysis, there was no significant difference among diets in terms of entropy accumulation rate (LEA-LP versus CON: p = 0.168, LEA-LP versus LEA-HP: p = 0.413, LEA-HP versus CON: p = 0.576). Each diet has the same effect on entropy generation and accumulation rates (see Table 9).

**Table 9:** Entropy accumulation rate and lifespan for three different diets.

	Annual entropy accumulation rate (kJ/year kg K)	Lifespan (year)
CON	162 ± 14 (95%Cl: 149–175)	72 ± 8 (95%Cl: 65–79)
LEA-LP	154 ± 13 (95%Cl: 142–166)	74 ± 7 (95%Cl: 68–80)
LEA-HP	154 ± 22 (95%Cl: 135–175)	73 ± 11 (95%Cl: 62–83)

The lifespan of the athletes was estimated based on entropy accumulation rate. For the situation of consumption of the diets throughout athletes’ lifespan, the longevity of athletes was 72 ± 8 years (95%Cl: 65–79), 74 ± 7 years (95%Cl: 68–80), and 73 ± 11(95%Cl: 62–83) for the CON, LEA-LP, and LEA-HP respectively. The diets influence lifespan estimation equally (LEA-HP versus CON: p = 0.763, LEA-HP versus LEA-LP: p = 0.212, CON versus LEA-LP: p = 0.343).

For the second (for 5 years of consumption of the LEA diets) and third conditions (for 10 years of consumption of the LEA diets), there is no significant difference among the diets in terms of entropy accumulation. Lifespan estimation was 89 ± 7(95%Cl: 81–96) years and 89 ± 8(95%Cl: 81–96) years for the CON diet, 89 ± 7(95%Cl: 82–96) years and 89 ± 772.3(95%Cl: 81–96) years for the LEA-LP diet, and 89 ± 8(95%Cl: 82–97) years and 89 ± 8(95%Cl: 81–96) years for the LEA-HP diet, respectively. Table 10 presents entropy accumulation until retirement and lifespan estimation for following the LEA diets for 5 and 10 years. There was no significant difference between the diets in terms of lifespan estimation (CON versus LEA-HP: p = 0.689, CON versus LEA-LP: p = 0.365, LEA-HP versus LEA-LP: p = 0.605 for 5 years of the LEA diets consumption and LEA versus LEA-LP: p = 0.689, CON versus LEA-LP: p = 0.366, LEA-HP versus LEA-LP: p = 0.605).

**Table 10:** Entropy accumulation and lifespan estimation for 5 and 10 years of the LEA diets and the lifespan increments for the second and third situations when compared to the first situation.

	Following the LEA diets for 5 years			Following the LEA diets for 10 years		
Diets	Entropy accumulation for 50 years (kJ/kg K)	Lifespan (year)	Increment in Lifespan (year)	entropy accumulation for 50 years (kJ/kg K)	Lifespan (year)	Increment in Lifespan (year)
CON	8090 ± 720 (95%Cl: 7424–8756)	89 ± 7 (95%Cl: 81–96)	17 ± 5 (95%Cl: 12–22)	8090 ± 720 (95%Cl: 7424.3–8755.8)	89 ± 8 (95%Cl: 81–96)	17 ± 5 (95%Cl: 12–22)
LEA-LP	8014 ± 698 (95%Cl: 7368–8659)	89 ± 7 (95%Cl: 82–96)	16 ± 6 (95%Cl: 11–22)	8052 ± 708 (95%Cl: 7306–8707)	89 ± 772.3 (95%Cl: 81–96)	16 ± 6 (95%Cl: 10–22)
LEA-HP	8020 ± 772 (95%Cl: 7306–8734)	89 ± 8 (95%Cl: 82–97)	17 ± 7 (95%Cl: 10–24)	8055 ± 744 (95%Cl: 7367–8743)	89 ± 8 (95%Cl: 81–96)	16 ± 8 (95%Cl:8–24)
Annual entropy accumulation for the retirement diet		87 ± 13 (95%Cl: 78–95)				

Lifespan estimation increased when athletes followed the LEA diets for only 5- and 10-years period, then continued the CON diet until their retirement. However, as shown in Table 9, the increment in lifespan estimation for the second and third states compared to the first did not differ.

## 4 Discussions

The purpose of this study was to determine thermodynamically how low energy availability impacts endurance-trained male athletes’ lifespans. Our primary finding was that although both LEA diets led to short-term energy deficiency, there was no significant difference among diets in terms of entropy generation/accumulation and lifespan estimation depending on macronutrient metabolism. Moreover, the diets did not differ from each other in terms of work performance.

### 4.1 Macronutrient metabolism rate changes with the diets

Daily macronutrient intake is significant for athletes' diets, and the composition of macronutrients changes depending on the diet type and energy demand. If energy intake with the diet does not meet energy demands, the athlete's body uses stored macronutrients [11,22]. Our results confirm that the type of diet affects the amount and percentage of macronutrients oxidized. Fat oxidation occurs to supply energy demand when glycogen is depleted, and glycogenesis occurs during a low-carbohydrate diet and fasting [52]. As protein intake was lowest during the LEA-LP diet, the percentage of protein metabolism was the highest, even though none of our subjects were in a negative protein balance (see Table S1). On the other hand, the LEA-LP diet increased the percentage of protein oxidation, and the percentage of carbohydrate metabolism was highest among athletes who consumed LEA-LP. The findings in Table 5 reveal a notable preference for carbohydrates over fat as the primary energy source during exercise, with carbohydrates contributing substantially more to energy release. This preference is particularly pronounced in the CON diet. However, despite differences in energy substrate utilization during exercise, the proportions of energy release from carbohydrates and fat among the various diets did not show significant variation. These results suggest a consistent metabolic response across different dietary interventions, emphasizing the dominant role of carbohydrates in meeting energy demands during exercise, irrespective of diet composition.

Table 6 illustrates the lipid reuse patterns observed during exercise across different dietary conditions. Remarkably, athletes on the CON diet did not exhibit any lipid reuse during exercise. However, when comparing the LEA diets, a notable difference emerged. Athletes on the LEA-HP diet demonstrated significantly higher lipid reuse during exercise compared to those on the LEA-LP diet. This suggests that despite both diets having the same energy availability, the higher protein content in the LEA-HP diet may enhance lipid mobilization and utilization during exercise. This could indicate a metabolic adaptation favouring lipid oxidation in the presence of higher protein intake, potentially impacting performance and recovery. Additionally, glycogen is “trapped” in the muscle and, therefore, must be oxidized during exercise because participants were in a CHO deficit; the exercise likely depleted muscle glycogen. Hence, there was no glycogen reuse observed during exercise for any of the diet groups, although our calculations suggested that there was glycogen reuse when considering total energy expenditure due to RMR, EEE, NEAT, and DIT. According to Poortmans and Carpentier (2016) [53], 0.4 mol equivalent of glucose can be formed from 110 g of mixed amino acids in the liver. The complete oxidation of leucine, isoleucine, and valine produces 43, 42, and 32 mol of ATP per mole, respectively. However, with a P/O ratio of 2.2 compared to 2.8 for fats and 3.1 for glycogen, amino acids are not efficient for maximum power production. In our study, during the LEA-LP diet, approximately 93% of the protein in the form of amino acids was oxidized, while 47.66% of them were oxidized throughout the LEA-HP diet. 52% of them were used during the CON diet. We assume that the remaining amino acids are used for the synthesis of new proteins, laying down new tissue, and cell-repairing [54].Low energy availability diets cause energy deficiency and low energy availability in athletes.

Our results indicate that both experimental diets with moderate-intensity exercise led to a state of energy deficiency and low energy availability. The LEA-LP and LEA-HP groups exhibited significant energy contributions from lipid reuse, reflecting a higher reliance on fat stores to meet energy demands. This substantial mobilization of fat stores led to weight loss and a reduction in fat mass as the body compensated for the energy deficit by utilizing stored lipids and glycogen. According to the literature, 62.76 (kJ/day) per kg FFM is considered inadequate for an athlete's health since energy availability is not adequate to meet the energy demand required to maintain physiological functions [41,55,56]. As has been shown in the previous study, short-term induction of all diets led to reductions in body mass (see Table S2). However, contrary to expectations, the weight difference among diets was not significant (p > 0.050). On the other hand, there was a significant difference between the LEA-HP and CON diets in terms of FM (p = 0.028).

Although low energy availability may negatively affect athletes' work performance through reduced muscle strength, increased fatigue, and other physical and physiological disorders [57], our results showed that LEA diets have similar effects on athletes’ work performance when compared to the CON diet. It is possible that the intervention duration was too short to observe potential negative impacts on work performance. Semerciöz et al. [20] defined “internal work” as the work performed by internal organs such as the brain, heart, lungs, liver, kidneys, etc., and “external work” as the observable work performance such as running, eating, walking, cycling, etc. Performing both internal and external work requires ATP utilization. Since ATP is utilized to achieve muscle work performance, energy release occurs with muscle work performance. The increase in work performance leads to a higher amount of ATP utilization, generating a higher amount of heat [37]. The energy release quantity depends on ambient temperature, food digestion, and muscular activity [58]. In this study, the factors that changed were energy and protein intake. Therefore, it may be asserted that metabolic energy release increases depending on calorie intake since the increment in food consumption increases in basal metabolic rate (BMR) [59]. On the other hand, there was no significant difference among the diets in terms of metabolic heat production.

### 4.2 Low energy availability diets and a control diet affect athletes’ lifespan similarly

According to Silva and Annamalai [46], fat and carbohydrates do not appreciably influence the entropy generation rate, while protein significantly affects entropy generation. The oxidizing agent acts as an electron acceptor, and the nutrient atoms' electrons are "shuffled" throughout the oxidation reaction. It was recently shown that the ratio between the electron mole transfer per mole is 4 times greater than the stoichiometric moles of O_2_. Since the higher heating value (HHV) per stoichiometric mole of O_2_ is 448 kJ and approximately constant for most fuel. During the complete reduction of oxygen in cellular respiration, electron mole transfer per mole equals 4 times stoichiometric O_2_ moles per mole fuel. Thus, each electron mole transfers 112 kJ [60,61]. HHV per stoichiometric mole of O_2_ does not vary significantly between carbohydrates and fats. For carbohydrates, the stoichiometric requirement is 6 mol of O_2_ per mole of carbohydrates, equating to 1 mol of O_2_ per unit caloric value (UCF). For fats like palmitic acid, the stoichiometric requirement is 23 of O_2_, or approximately 1.44 mol of O_2_ per UCF. In the case of protein oxidation, 5.7 mol of O_2_ are required, translating to 1.25 mol of O_2_ per UCF protein. The electron transfer per UCF is highest for fats, with 23 electron moles transferred per mole of fat [62]. Therefore, the connection between electron transfer and inefficient oxidation of proteins underscores the impact of nutrient metabolism on oxidative stress and cellular health. Furthermore, low metabolic efficiency in P oxidation results in higher entropy generation compared to carbohydrates and fats. In the current study, in the case of the restricted energy intake during LEA-LP and LEA-HP diets, the reuse of glycogen and lipids (see Table S1) to meet energy requirements led to entropy generation. However, there was no significant difference in entropy generation rate among diets, as the amount of protein oxidation was similar (Fig. 4). When energy accumulates in the body, temperature increases, and so does entropy. Therefore, entropy, being a property of accumulation, arises from the changing temperature within the body. However, homeostatic mechanisms work to keep the body temperature at a certain point.

An increase in entropy accumulation rates is associated with a decrease in lifespan. When the body generates more entropy than it exports, the entropy accumulation rate within the body increases, leading to aging and damage [35]. The human body cannot export generated entropy with 100% efficiency [35]. This study proves this; according to our results, entropy generation was higher than entropy exportation for all diets. Entropy was exported at approximately 99%, with heat loss in all cases (see Table S4). Considering that the average lifespan of humans in Europe was 79 years as of 2019, according to Our World in Data (https://ourworldindata.org/life-expectancy, accessed on January 20, 2023) [63], all diets in the first condition reduced the estimated average lifespan by increasing entropy accumulation rates. Calculations based on oxygen and carbon dioxide estimation serve as indicators of the extent of oxidation of carbohydrates and fats. If the energy expenditure surpasses the intake for LEA, stored glycogen and lipids become pivotal in meeting the energy demands. A low energy availability diet provoked the reuse of glycogen and lipids, which caused more entropy accumulation. CON diet also led to excess energy intake and, thus, increased accumulation rates. Therefore, the lifespan estimation of the athletes decreased for all cases (Fig. 4). However, our results suggested that lifespan estimation increases when the LEA diets are consumed only for 5 and 10 years (shown in Table 10). Both diet periods affect the lifespan estimation similarly.

Building upon the previous study [19], significant differences were noted in hormonal responses such as insulin-like growth factor (IGF-1) and leptin, along with changes in bone turnover markers (P1NP and CTX-I) between the control and LEA diets. The alterations in bone turnover markers during LEA-LP and LEA-HP indicated that a shift favoured bone resorption. Interestingly, in contrast to these findings, our study did not reveal any notable distinctions in entropy generation rates and lifespan across different dietary settings. These results suggest that LEA diets and varied protein content may not noticeably impact athletes’ entropy generation rates and lifespan within the studied timeframe, whereas they considerably triggered hormonal and bone turnover alterations. The consideration of bone metabolism is pivotal in the analysis of lifespan and "health span." Prolonged bone resorption beyond the age of 35, including the post-menopausal phase in women, leads to diminished bone density. This decrease significantly elevates the risk of fractures and premature mortality [64].

The life history theory explains how the human body strategically allocates limited energy resources across various biological functions, highlighting “trade-offs,” where energy directed to support one function may compromise others due to scarcity [65]. For athletes experiencing LEA, this provides a relevant framework. This theory predicts intensified trade-offs among biological processes under conditions of scarcity, especially pertinent to athletes facing LEA challenges. The intricacies of these energetic trade-offs are influenced by multifaceted factors such as gender, life stage, training regimen, health status, dietary composition, and the duration and severity of exposure to LEA.

Lewis et al., 2023 shed light on the adaptability of athletes to ‘LEA, emphasizing the diversity in their physiological responses based on unique adaptation capabilities [66]. This variability underscores the complexity of the relationship between LEA and metabolic adaptation, emphasizing the need for individualized approaches in understanding and addressing the impacts of limited energy availability among athletes. The human body's ability to modulate energy expenditure (EE) rates metabolically to mitigate the effects of energy deficiency further emphasizes the intricate mechanisms underlying physiological responses to LEA. This adaptability further underscores the significance of considering individual differences when evaluating the impact of LEA on athletes' overall health and performance [5].

In conclusion, similarities between entropy accumulation and estimated lifespan dependent on CON and LEA diets might result from metabolic adaptation to energy deficiency.

### 4.3 Limitations of the study

There were several limitations related to the study. We had neither 24-h oxygen consumption, carbon dioxide production amount, nor urinary nitrogen level; therefore, we had to make some assumptions to calculate the oxidation rate of macronutrients. There were no data associated with pre-experiment energy and food intake. Moreover, the duration of the experiments was short, and we did not have knowledge of the physiological effects of a long-term LEA. In this study, we designed a model to predict its further effect on entropy accumulation and lifespan estimation. Hence, the entropy generation and accumulation rate pre-experiment was not calculated. Moreover, a larger sample size can enable results that are more accurate. The results of the current study should be evaluated carefully since athletes’ muscular development, mental health, and further physiological state were not considered during this study. On the other hand, our results help to understand how energy intake and the type of diet under the influence of exercise would affect the athletes' entropy generation rate and lifespan. This study serves as a guide for further research related to athlete health and performance.

## 5 Conclusion

In this study, our thermodynamic analysis played a crucial role in assessing the impact of short-term low energy availability (LEA) on the estimated lifespan of endurance-trained athletes. Despite variations in energy deficiency and availability across diets, the thermodynamic perspective revealed no significant differences in lifespan estimations (CON: 72 ± 8 years, LEA-LP: 74 ± 7 years, LEA-HP: 73 ± 11 years). The influence of protein intake on these outcomes was inconclusive. This study underscores the importance of incorporating thermodynamic considerations in lifespan estimation studies, providing valuable insights into the intricate relationship between energy availability, entropy accumulation, and the overall well-being of athletes. However, further research with expanded sample sizes, varied exercise intensities, and consideration of factors like stress, lifestyle, and sleep quality is needed to deepen our understanding of the impact of LEA diets on physiological processes and their implications for athletes' health and performance.

**Table:** Nomenclature

ATP	Adenosine Triphosphate
Cl	Confidence Interval
CON	Control diet group
CR	Caloric Restriction
DIT	Dietary-Induced Thermogenesis
EA	Energy Availability
EB	Energy Balance
EE	Exercise Energy Expenditure
EE	Energy Expenditure
EI	Energy Intake
FM	Fat Mass
FFM	Fat-Free Mass
kJ	Kilojoule
kJ/kg K	Kilojoules per kilogram per Kelvin
kW	Kilowatt
kW/kg K	Kilowatts per kilogram per Kelvin
LEA	Low Energy Availability
LEA-HP	Low Energy Availability High Protein diet group
LEA-LP	Low Energy Availability Low Protein diet group
NEAT	Non-Exercise Activity Thermogenesis
${\dot{Q}}_{total}$	Heat Transfer from the body due to perspiration and respiration
RMR	Resting Metabolic Rate
S_gen_	Entropy Generation
S_acc_	Entropy Accumulation
T_b_	Human Body Temperature (37 °C)
W_ATP_	Work done by ATP production
TEE	Total Energy Expenditure
UCF	Unit Carbon Formula
VO_2_	Volume of Oxygen Consumption
VCO_2_	Volume of Carbon Dioxide Production
ΔH	Change in Enthalpy
ΔS	Change in Entropy
ΔT	Change in Temperature

## Funding statement

This research did not receive any specific grant from funding agencies in the public, commercial, or not-for-profit sectors.

## Data availability statement

Data included in article/supp. material/referenced in the article.

## CRediT authorship contribution statement

**Cennet Yildiz:** Writing – original draft, Visualization, Validation, Methodology, Investigation, Formal analysis, Conceptualization. **Karsten Köhler:** Writing – review & editing, Validation, Supervision, Resources, Project administration, Methodology, Formal analysis, Data curation. **Paulina Wasserfurth:** Writing – review & editing, Validation, Investigation, Data curation. **Mustafa Özilgen:** Writing – review & editing, Validation, Supervision, Methodology, Formal analysis.

## Declaration of generative AI and AI-assisted technologies in the writing process author-disclosure

Statement: During the preparation of this work the author(s) used Grammarly/Grammarly AI tool in order to editing and spelling check. After using this tool/service, the author(s) reviewed and edited the content as needed and take(s) full responsibility for the content of the publication.

## Declaration of competing interest

The authors declare that they have no known competing financial interests or personal relationships that could have appeared to influence the work reported in this paper.
