# Supplementary material for: Assessment of entropy accumulation in human subjects when exposed to low energy availability
Source: Heliyon. 2024 Aug 28;10(17):e36792. doi: 10.1016/j.heliyon.2024.e36792 (PMC11402755; doi:10.1016/j.heliyon.2024.e36792)
Supplement: Multimedia component 1 [file mmc1.docx]

**SUPPLEMENTARY MATERIAL**

**
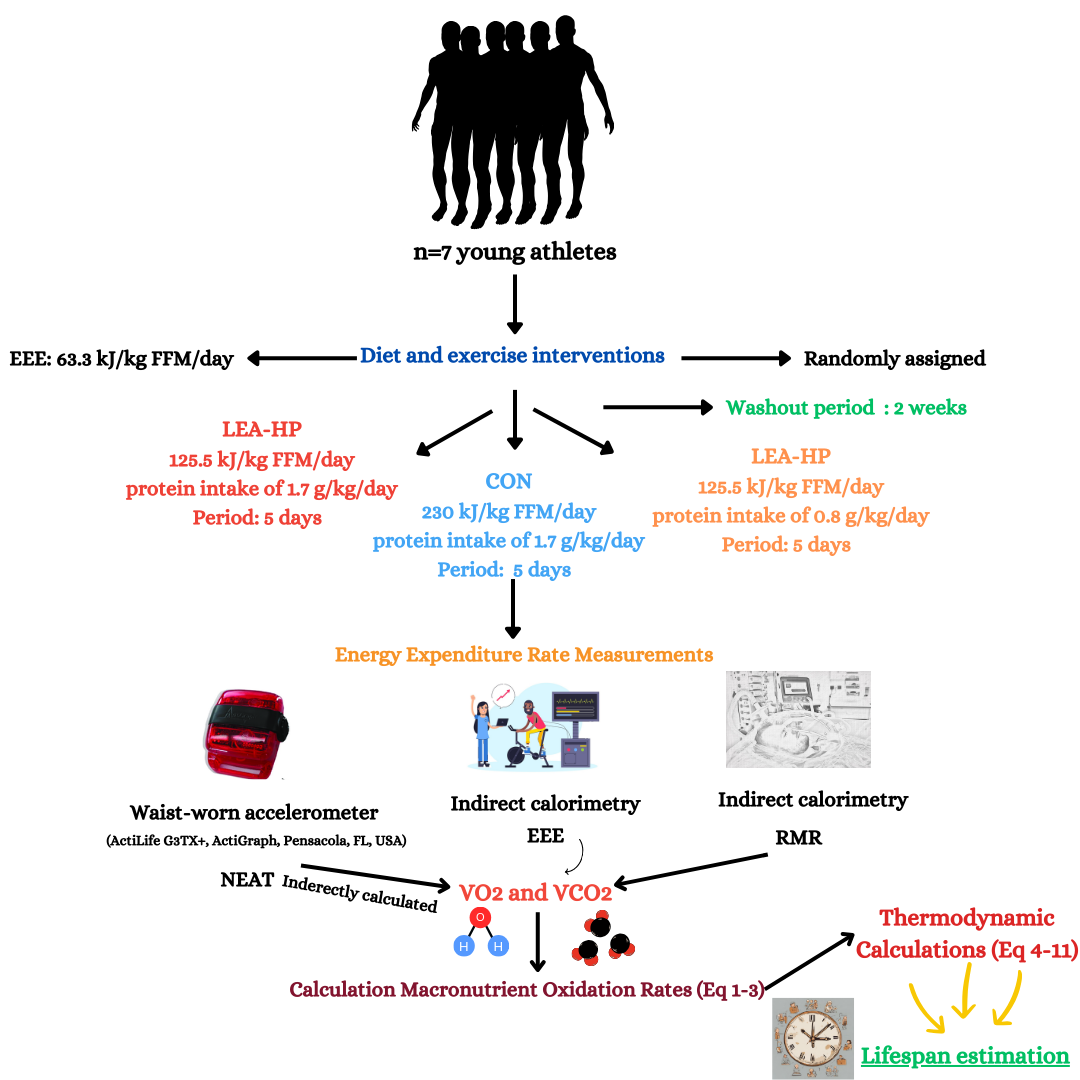
**

**Figure S1**: Schematic description of the study design


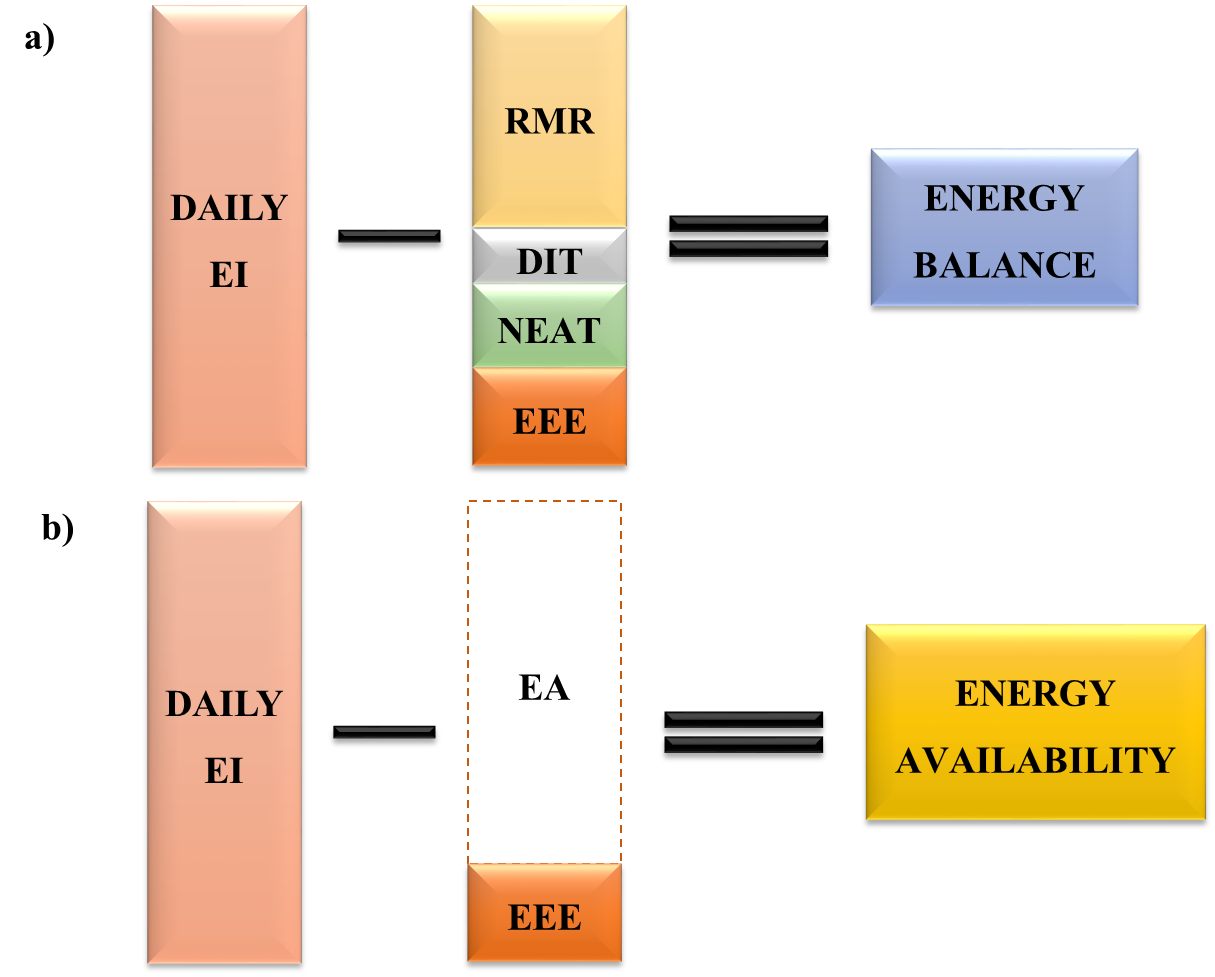


**Figure S2:** Schematic description for energy conversion: **a)** energy balance and **b)** energy availability.

**Table S1.** Macronutrient balance for each type of diet

|  | **Protein storage (g/d)** | **Carbohydrate storage**  **(g/d)** | **Fat storage**  **(g/d)** | **Glycogen reuse (g/d)** | **Lipid reuse (g/d)** |
| --- | --- | --- | --- | --- | --- |
| CON | 69.56 ± 14.13 (95%Cl: 54.49-82.63) | 116.29±158.4  (95%Cl: -30. 34-262.92) | 49.57 ± 88.00  (95%Cl: -31.89-131.04) | 116.06 ±177.43  (95%Cl: -70.13-302.26) | 68.69 ± 90.50  (95%Cl: -26.6-163.68) |
| LEA-LP | 4.41 ± 7.25  (95%Cl: -2.30-11.2) | 72.11±69.34  (95%Cl: 7.98+136.24) | 0 | 27.46 ± 55.99  (95%Cl: -19.35-74.28) | 126.83 ± 94.99  (95%Cl: 47.41-206.25) |
| LEA-HP | 71.18 ± 34.02 (95%Cl: 39.71-102.65) | 11.31±29.94  (95%Cl: -16.37+39.00) | 0 | 195.65 ±160.37  (95%Cl: 47.32-343.97) | 142.37 ± 109.84  (95%Cl: 40.77-243.95) |

Protein, carbohydrate, and fat storage and glycogen and lipid reuse were calculated by using macronutrient intake (presented in Table 2) and macronutrient metabolization/oxidation data (computed by Eq.1-3). These data represent the mean and standard deviation and are not valid for each participant. Glycogen and lipid reuse were not the case for all participants.

**Table S2.** Difference for FM, FFM, and weight before and after the study.

|  | **FM (kg)** | **FFM (kg)** | **Weight (kg)** |
| --- | --- | --- | --- |
| CON | 0.31±2.03  (95%Cl: -1.57-2.20) | -0.33±2.31  (95%Cl: -2.46-1.81) | -1.53±1.54  (95%Cl: -2.95-(-)0.10) |
| LEA-LP | -0.81±0.99  (95%Cl: -1.74-0.11) | -1.31±1.44  (95%Cl: -2.64-0.14) | -2.12±0.80  (95%Cl: -2.88-(-)1.40) |
| LEA-HP | -2.35±3.00  (95%Cl: -5.50-0.80) | 0.15±2.30  (95%Cl: -2.25-2.55) | -2.20±1.43  (95%Cl: -3.71-(-)0.69) |

The discrepancies in the parameters were computed based on the pre-and post-data for each participant. A negative sign (-) denotes decreases in FM, FFM, and weight. There was a significant difference between the LEA-HP and CON diets with regard to FM (p=0.028), while FFM and weight did not show variance across the diets (p>0.050).

**Table S3.** Metabolic energy release and heat loss due to respiration and perspiration with each type of diet

|  | **Metabolic energy release (kW/d)** | **Heat loss (kW/d)** |
| --- | --- | --- |
| CON | 175.37 ± 33.56  (95%Cl: 144.42-206.41) | 149.44 ± 67.31  (95%Cl: 87.18-211.76) |
| LEA-LP | 146.76 ± 15.94  (95%Cl: 132.05-161.46) | 141.57± 15.88  (95%Cl: 127.59-155.56) |
| LEA-HP | 148.53 ± 19.93  (95%Cl: 130.19-167.87) | 144.83 ± 19.76  (95%Cl: 127.14-162.43) |

**Table S4.** Annual entropy generation and exportation rate for each diet

|  | **Total entropy generation rate**  **(kW/ year kg K)** | **Entropy exportation rate (kW/year kg K)** | **% Entropy exported via advection** | **% Entropy via heat loss** |
| --- | --- | --- | --- | --- |
| CON | 5.36 x 10 ^-6^ ± 5.21 x 10 ^-7^  (95%Cl: 4.87 x 10 ^-6^ -5.83 x 10 ^-6^ ) | 2.45 x 10^-7^ ± 2.13 x 10^-8^  (95%Cl: 2.25 x 10^-7^-2.65 x 10^-7^) | 0.47 ± 1.24  (95%Cl: -0.65-1.65) | 99.50 ± 1.24  (95%Cl: 98.35-100.65) |
| LEA-LP | 5.17 x 10 ^-6^ ± 4.74 x 10 ^-7^  (95%Cl: 4.73 x 10 ^-6^ -5.61 x 10 ^-6^ ) | 2.33 x 10^-7^ ± 1.99 x 10^-8^  (95%Cl: 2.05 x 10^-7^-2.65 x 10^-7^) | 0.32 ± 0.7  (95%Cl: -0.32-0.98) | 99.68 ± 0.04  (95%Cl: -99.02-100.32) |
| LEA-HP | 5.30 x 10 ^-6^ ± 7.01 x 10 ^-7^  (95%Cl: 4.65 x 10 ^-6^ -5.95 x 10 ^-6^ ) | 2.35 x 10^-7^ ± 3.28 x 10^-8^  (95%Cl: 2.15 x 10^-7^-2.52 x 10^-7^) | 0.02 ± 0.003  (95%Cl: 0.14-0.2) | 99.98 ± 0.004  (95%Cl: 99.979-99.986) |

**Table S5.** Total calories and composition of the diet plan for a 50-year-old healthy retired athlete

| **Total calorific uptake**  **(kcal/kg per day)** | **A 50 year- old healthy retired athlete’s diet composition** | **% of calories from macronutrients** |
| --- | --- | --- |
| Total calorific uptake (kcal/kg per day) | 2000 |  |
| Carbohydrate (g/kg day) | 280 | 56% |
| Protein (g/kg day) | 77 | 15.4% |
| Fat (g/kg day) | 66 | 29.7% |
